# The Clinical Spectrum of Inflammatory Bowel Disease Associated With Specific Genetic Syndromes: Two Novel Pediatric Cases and a Systematic Review

**DOI:** 10.3389/fped.2021.742830

**Published:** 2021-10-26

**Authors:** Simona Gatti, Giulia Gelzoni, Giulia N. Catassi, Carlo Catassi

**Affiliations:** ^1^Department of Pediatrics, Polytechnic University of Marche, G. Salesi Children's Hospital, Ancona, Italy; ^2^Department of Maternal and Child Health, Pediatric Gastroenterology and Liver Unit, Umberto I Hospital, Sapienza University of Rome, Rome, Italy

**Keywords:** inflammatory bowel disease, Crohn disease, ulcerative colitis, Down syndrome, Turner syndrome, genetic syndromes, neurofibromatosis, DiGeorge syndrome

## Abstract

**Background and Aims:** Inflammatory bowel disease (IBD) is a typical polygenic disorder and less frequently shows a monogenic origin. Furthermore, IBD can originate in the context of specific genetic syndromes associated with a risk of autoimmune disorders. We aimed to systematically evaluate the prevalence of IBD in specific genetic syndromes and to review the clinical characteristics of the published cases.

**Methods:** According to the Preferred Reporting Items for Systematic Reviews and Meta-Analyses (PRISMA) guidelines, studies describing patients with IBD and a genetic syndrome and/or studies indicating the prevalence or incidence of IBD in subjects with a genetic syndrome were included.

**Results:** Forty-six studies describing a total of 67 cases of IBD in six genetic syndromes and two personally assessed unpublished cases were included in the review. The majority of cases were associated with Turner syndrome (TS) (38 cases), Down syndrome (DS) (18 cases) and neurofibromatosis type 1 (NF1) (8 cases). Sporadic cases were described in DiGeorge syndrome (2), Kabuki syndrome (2), and Williams syndrome (1). The prevalence of IBD ranged from 0.67 to 4% in TS and from 0.2 to 1.57% in DS. The incidence of IBD was increased in TS and DS compared to the general population. Eight cases of IBD in TS had a severe/lethal course, many of which described before the year 2000. Two IBD cases in DS were particularly severe.

**Conclusion:** Evidence of a greater prevalence of IBD is accumulating in TS, DS, and NF1. Management of IBD in patients with these genetic conditions should consider the presence of comorbidities and possible drug toxicities.

**Systematic Review Registration**: PROSPERO, identifier: CRD42021249820

## Introduction

Inflammatory bowel disease (IBD) includes a group of chronic intestinal inflammatory conditions with three main clinical subtypes: Crohn's disease (CD), ulcerative colitis (UC), and IBD-unclassified (IBD-U). IBD has a multifactorial pathogenesis, involving genetic susceptibility, environmental factors, intestinal microbiota, and immune system. The contribution of genetic factors has been extensively described; however, the impact of genetics in the development of IBD is variable. The most common form of IBD is a polygenic disorder where the implication of genetic factors can significantly impact on the susceptibility and/or in the development of specific clinical characteristics and complications of the disease (1). So far, more than 230 disease loci related to polygenic IBD have been defined by genome-wide association studies (GWAS) (2). Differences between IBD subtypes, associations with clinical features, and prognosis can be explained, at least partially, by GWAS (3). Conversely, fewer common variants in IBD risk loci have been discovered to have a causative role including NOD2, ATG16L1, IRGM, IL23R, CARD9, RNF186, and PRDM1, with most risk loci containing different candidate genes (4, 5). More recently, monogenic forms of IBD have been identified as a group of diseases characterized by an early onset (generally in children <10 years of age: EO-IBD or very early in children <6 years of age: VEO-IBD) and a severe course, refractory to conventional therapies. More than 70 monogenic defects have been described, with a considerable number of the implicated genes involved in the maintenance of the immune homeostasis (6).

**Figure 1:**
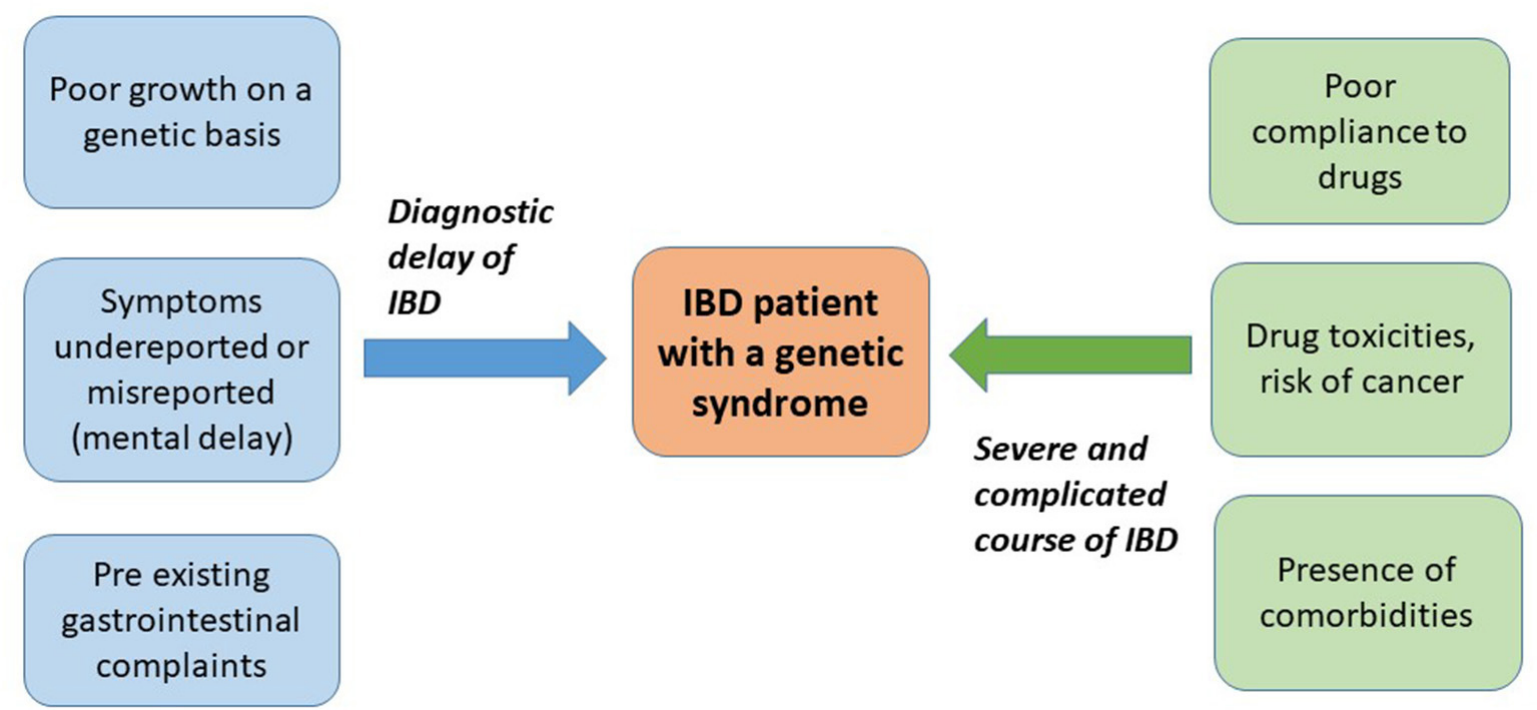
Peculiarities of inflammatory bowel disease (IBD) diagnosis and course in the context of a genetic syndrome.

In addition to these groups of IBD (polygenic and monogenic forms), some genetic syndromes, mainly characterized by chromosomal abnormalities (numerical or structural), can present with IBD or are at high risk of development of IBD. These syndromes can be associated with autoimmune comorbidities, with the usual paradigms being represented by Turner syndrome (TS) and trisomy 21 or Down syndrome (DS). IBD has been reported, although sporadically, in other genetic syndromes. Possible mechanisms to explain the risk of autoimmunity in such syndromes include the following: genetic defects of immune regulatory pathways, impaired apoptosis and defense against oxidative stress, and molecular mimicry of viral or bacterial antigens (7). Clinical course of IBD in patients with a genetic syndrome can substantially differ from non-syndromic IBD for several reasons, including the following: the intellectual disability, the difficulties in adherence to treatment, a different response to certain medications, and the presence of comorbidities (Figure 1). So far, no specific protocols or guidelines exist for such vulnerable patients.

We aimed to describe this specific scenario starting by illustrating two challenging pediatric cases of IBD in subjects with a genetic syndrome. We further decided to perform a systematic review of literature to identify the frequency of IBD in specific syndromes and to describe the clinical characteristics of IBD in patients with these genetic conditions.

### Case Report 1

A 3-year-old Italian girl presented to our attention with a 4-week history of bloody diarrhea. She was born from non-consanguineous Italian parents; her grandfather was affected by IBD, and her mother was followed up for mild thrombocytopenia and neutropenia of unknown origin. At the time of admission, she was clinically well and her weight and height were both below the 3rd centile. Blood tests were unremarkable. Stool culture and examination for fecal parasites were negative. Fecal calprotectin was mildly elevated (245 mg/kg) and p-ANCAs (anti-neutrophil cytoplasmic antibodies) were positive. Ileo-colonoscopy revealed pancolitis with edema, signs of inflammation, and tendency to bleeding along the entire colon. Histological findings showed mostly acute inflammation with minimal signs of chronicity. In the suspicion of UC, she was started on oral mesalazine with clinical remission achieved after 2 weeks. At the 3-month follow-up, she was further investigated for her persistent failure to thrive with short stature and retarded growth velocity. Mild dimorphisms were noted at that time. Atrial septal defect was found at echocardiogram. Chromosomal analysis indicated a Turner syndrome (mosaic pattern, 96% 45,X and 4% 46,XX). Five months later, she presented recurrence of gastrointestinal symptoms (bloody diarrhea) and raised fecal calprotectin of 350 mg/kg. She was endoscopically re-evaluated, and the examination showed hyperemia and edema of colic mucosa with mild ulcers in the sigmoid and transverse colon and normal appearance of the terminal ileum. Histology showed signs of acute and chronic colitis with infiltration of lymphocytes, eosinophilic, and neutrophil granulocytes; aphthous erosions; and focal alteration of glandular architecture along the ascending, transverse, and descending colon. The data confirmed a diagnosis of UC. She was started on oral steroids and azathioprine, and clinical and laboratory remission was achieved. In the following months, she required another course of steroids. A year later, the patient was admitted for an endoscopic re-evaluation, and the colonoscopy showed mild hyperemia and edema (Mayo score 1). Treatment with azathioprine was continued. At the age of 4, she started growth hormone (GH) therapy. Currently, she is 6 years old and she maintains clinical remission with azathioprine alone (2.6 mg/kg/day).

### Case Report 2

A 10-year-old boy of Arab origin with DS was admitted to our institution for a 3-week history of fever, diarrhea, and erythema nodosum. In the preceding 4 years, he had been complaining of dysphagia for solid foods, feeding on only creamy foods and liquids. At the time of our evaluation, his chemical panel revealed the following: normocytic anemia, low albumin, raised C-reactive protein (CRP) and erythrocyte sedimentation rate (ESR), and raised fecal calprotectin (>500 mg/kg). Anti-Saccharomyces cerevisiae antibodies (ASCA) were present. Stool cultures were negative. An endoscopic examination showed a proximal esophageal stricture (with mild signs of mucosal inflammation), edema, and aphthous ulcers in the rectum, descending, and transverse colon. Histological features of the colic and rectal mucosa included severe infiltration of lymphocytes and eosinophilic granulocytes and rare crypt abscesses; in the sigmoid colon, there was an epithelioid granuloma. From these findings, a diagnosis of CD was made. In consideration of the mild signs of inflammation and the proximal site of the esophageal stricture (not typical for CD), further etiologies, different from CD, were considered, including a previously not reported caustic ingestion and a congenital stenosis, although the appearance at 6 years of age was less likely. Treatment of CD was started with exclusive enteral nutrition (EEN) associated to anti-tumor necrosis factor (TNF) therapy (infliximab 5 mg/kg). Clinical remission was initially achieved. Three months after the initial treatment, he was admitted again for fever; diarrhea; and reappearance of erythema nodosum, asthenia, and weight loss. Abdominal ultrasound showed wall thickening (5–6 mm) of the descending and sigmoid colon and rectum. Azathioprine and steroid therapy was initiated, and the infusion of infliximab was anticipated (every 6 weeks) at a higher dose (7 mg/kg). Five months later, another hospitalization was necessary following an episode of impact of the bolus, dysphagia, and diarrhea with blood. An endoscopic dilation of the esophageal stricture was performed. The abdominal ultrasound revealed a worse wall thickening of the descending and sigmoid colon and rectum (12 mm) and a thickening of the terminal ileum, ileocecal valve, cecum, and ascending colon (7 mm). Another course of EEN was commenced, treatment with infliximab was optimized (every 4 weeks at the dose of 10 mg/kg), and azathioprine was continued. Almost a year after the diagnosis, the patient was endoscopically re-evaluated. A Savary dilation of the esophagus was repeated; the colonoscopy showed hyperemia, edema, ulcers, and fibrin deposition in the sigmoid colon and rectum. Histological findings revealed a chronic inflammation of the esophagus (with signs of fibrosis), stomach, cecum, colon, and rectum. Six months later, he required another esophageal endoscopic dilation, for an episode of impact of bolus. Due to the persistence of clinical activity and raised inflammatory markers (including CRP, ESR, and calprotectin), maintenance therapy with infliximab and azathioprine was switched to adalimumab and azathioprine and another course of oral steroids was required. At the 2-month follow-up, the patient is in clinical remission.

## Materials and Methods

### Registration and Search Strategy

The study was registered in the PROSPERO database registry (PROSPERO registration number CRD 42021249820). The medical literature was searched using MEDLINE, EMBASE, and the Cochrane Central Register of Controlled Trials, until December 2020, according to the Preferred Reporting Items for Systematic Reviews and Meta-Analyses (PRISMA) guidelines (8, 9). A separate systematic research was performed for the following syndromes: Turner syndrome or monosomy X, Down syndrome or trisomy 21, Klinefelter syndrome or XXY syndrome, Edward syndrome or trisomy 18, Patau syndrome or trisomy 13, Williams syndrome, Noonan syndrome or RASopathies, Prader Willi syndrome, Angelman syndrome, DiGeorge syndrome or 22q deletion syndrome, Kabuki syndrome, and neurofibromatosis or von Recklinghausen's disease. The genetic conditions were chosen considering the most frequent chromosomal abnormalities and syndromes with a known risk of autoimmune disorders. A non-specific research was also performed using the term “genetic syndrome” instead of a specific name. For each syndrome, the different names of the condition were combined with “Inflammatory Bowel Disease” OR “IBD” OR “ulcerative colitis” OR “Crohn.” A separate search was performed matching each syndrome name or the generic term “genetic syndrome” AND the terms “autoimmune OR autoimmunity.”

### Inclusion Criteria, Data Extraction, and Outcome Assessment

We included all the prospective controlled trials, observational studies, case–control studies, case reports, and series describing cases of IBD (both in children and adults) or studies reporting epidemiological data (prevalence or incidence) of IBD in a population of subjects with a genetic syndrome. Due to the paucity of data, we also included letters, comments, and review articles. We excluded non-English literature. Two independent reviewers (SG and GG) performed the first selection, screened title and abstract of the papers identified by electronic search, and completed an inclusion form for eligible studies. Additional articles were obtained through citation snowballing to locate primary sources. When no abstract was available, the article was always screened by full text. The same two authors read in full all the selected articles, and disagreements were discussed and resolved with the aim of two further authors (GNC and CC). The prevalence or incidence of IBD in a specific syndrome was extracted from the cohort studies, whenever available. Clinical characteristics, medical and surgical treatments, and outcomes of subjects with IBD and a genetic syndrome were summarized. The description of our two cases was performed, following parental consent.

## Results

**Figure 2:**
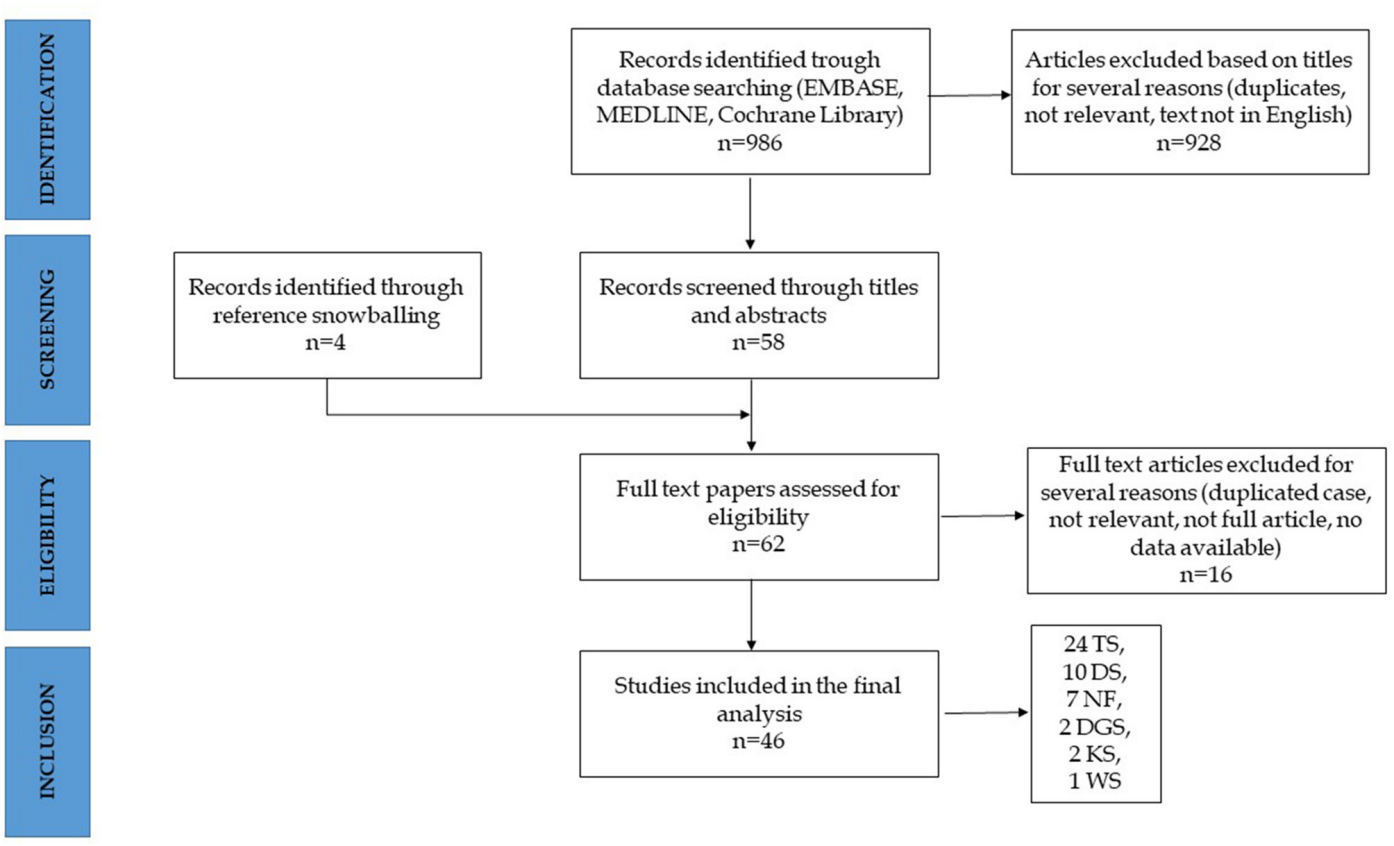
Algorithm of inclusion. TS, Turner's syndrome; DS, Down syndrome; NF, neurofibromatosis; DGS, DiGeorge syndrome; KS, Kabuki syndrome; WS, Williams syndrome.

The systematic research identified a total of 986 articles, of which 58 were considered relevant based upon the established inclusion criteria (selection through title and abstract). Furthermore, four papers were identified through reference snowballing. A total of 62 full-text articles were assessed for eligibility and, after accurate reading, 46 were included in the final review. Sixteen articles were excluded, and the reasons for exclusion were as follows: duplicates (reporting cases described elsewhere), not reporting cases of IBD, or not describing either clinical or epidemiological aspects (Figure 2). The 46 articles reported a total of 67 cases of IBD in 6 different syndromes: 37 in TS, 17 in DS, and 13 in other syndromes including 2 cases in DiGeorge syndrome (DGS), 8 cases in neurofibromatosis type 1 (NF1), 2 cases in Kabuki syndrome (KS), and 1 case in Williams syndrome (WS).

### Clinical Features and Prevalence of IBD in Turner Syndrome

A total of 24 papers describing cases or reporting epidemiological data of IBD in TS were included. Table 1 illustrates the characteristics of all the 24 studies (10–33). Thirty-seven cases of TS and IBD (17 with CD, 14 with UC, and 6 cases named as IBD) were reported by 21 articles (10–24, 26–29, 32, 33). A detailed clinical description was available for 25 cases only (10–21, 24, 26–29, 33). Table 2 summarizes the clinical features of the published cases and the unpublished cases presented in this article. Thirteen (50%) were pediatric cases (7 CD, 5 UC, and 1 IBD) (11, 12, 14–16, 18, 21, 27, 28).

**Table 1:** Characteristics of published studies reporting cases of IBD in TS subjects.

**References**	**Publication year**	**Study design**	**Population (*n*)**	**IBD cases**	**Prevalence of IBD in TS**
Williams et al. (10)	1966	Case report	1 subject	1 patient with UC	
Weinrieb et al. (11)	1976	Case series	2 subjects	1 patient with CD 1 patient with UC	
Keating et al. (12)	1978	Case report	1 subject	1 patient with CD	
Scobie et al. (13)	1979	Case report	1 subject	1 patient with UC	
Price et al. (14)	1979	Case series	135 patients with TS observed over an average period of 11 years	2 patients with CD 2 patients with UC	Annual rate of IBD in TS: 270/100,000 In the series of 135 TS, the prevalence of CD and UC was 1.48%, compared with the general population; 30-fold increased and 16-fold increased
Arulanantham et al. (15)	1980	Case series	5 patients with TS and IBD (2 reported in literature and 3 new cases)	1 patient with UC 2 patients with CD	
Kohler et al. (16)	1981	Case series	2 subjects	2 patients with CD	
Nishimura et al. (17)	1985	Case report	1 subject	1 patient with UC	
Knudtzon et al. (18)	1988	Case report	1 subject	1 patient with CD	
Knudtzon et al. (19)	1988	Case report and review of literature	17 TS with IBD reported in literature + 1 new case	1 patient with CD	
Manzione et al. (20)	1988	Case report and review of literature	17 patients with TS and IBD (16 reported from literature + 1 new case)	1 patient with UC	
Lacaille et al. (21)	1995	Case report	1 subject	1 patient with IBD	
Hayward et al. (22)	1996	Review	15 patients with TS and IBD since 1966 (14 reported in literature + 1 new case)	1 patient with CD	
Gravholt et al. (23)	1998	Cross-sectional study	594 females with TS	4/594 with IBD	Prevalence of IBD in TS: 0.67%
Sokol et al. (24)	2002	Case report	1 subject	1 patient with IBD	
Elsheikh et al. (25)	2002	Review	270 women with TS		Prevalence of IBD of 2.6%
Durusu et al. (26)	2005	Case report	1 subject	1 patient with CD	
Takaya et al. (27)	2006	Case report	1 subject	1 patient with UC	
Hyodo et al. (28)	2009	Case report	1 subject	1 patient with UC	
Triantafillidis et al. (29)	2010	Case report	1 subject	1 patient with CD	
Jorgensen et al. (30)	2010	Cross-sectional study	798 women with TS		SIR (standardized incidence ratios) UC: 2.5 (1.2–4.7); CD: 1.5 (0.4–3.9)
Bakalov et al. (31)	2012	Prospective study	224 women with TS		Prevalence of IBD in TS: 4%
Hanew et al. (32)	2018	Cross-sectional study	385 patients with TS	7/385 with IBD (1.8%): 4/385 with UC (1%) and 3/385 with CD 0.8%	Prevalence of IBD in TS: 1.8% (0.8% CD, 1 % UC) vs. the prevalence of IBD in general population: 0.03% CD, 0.1% UC
Keating et al. (33)	2020	Case report	1 subject	1 patient with CD	

**Table 2:** Clinical characteristics of the cases of IBD and TS detailed in literature.

**Reference, year**	**Clinical characteristics**	**Treatment**	**Karyotype**
Williams et al. (10), 1966	1 patient (40-year-old female) with UC. She died after surgery for heart attack	Steroids + colectomy	X0/X iso X mosaic
Weinrieb et al. (11), 1976	1 patient (17-year-old female) with CD (sigmoid and transverse colon) 1 patient (41-year-old female) with UC. Died after operation of a cardiorespiratory arrest	Intermittent courses of steroidsParenteral steroids, colectomy, and ileostomy	45 X/46,X,i(Xq) 45X/46,X,i(Xq)
Keating et al. (12), 1978	1 patient (17-year-old female) with CD (ileum; fistula ileum-bladder)	Total parenteral nutrition; surgery	45X0
Scobie et al. (13), 1979	1 patient (20-year-old female) with celiac disease and UC	Prednisone	46X/IsoX
Price et al. (14), 1979	1 patient (35-year-old female) with CD 1 patient (26-year-old female) with UC (transverse, descending, and sigmoid colon and rectum) 1 patient (19-year-old female) with CD. She died after operation 1 patient (16-year-old female) with UC (fistula in the right iliac fossa)	SteroidsSteroid retention enemas; sulfasalazineSurgery (cecostomy)Sulfasalazine, steroids, proctocolectomy, and ileostomy	45,X 46,X,i(Xq) 45,X/46,Xi(Xq) 46,X,del(X)(p)
Arulanantham et al. (15), 1980	1 patient (13-year-old female) with UC 1 patient (9-year-old female) with CD 1 patient (14-year-old female) with CD	ColectomyMedical treatmentSulfasalazine	30% 45X, 70% 46XX 45,X/46,X,i(Xq)/ 47,X,i(Xq),i(Xq) 45,X
Kohler et al. (16), 1981	1 patient (15-year-old female) with CD (colon) 1 patient (15-year-old female) with CD (fistula in the distal ileum-bladder); she died after operation because of tetanus	SulfasalazineHemicolectomy	46Xi(Xq) 45X/46XX
Nishimura et al. (17), 1985	1 patient (33-year-old female) with UC and Hashimoto thyroiditis		46,X,i(Xq)
Knudtzon et al. (18), 1988	1 patient (8.5-year-old female) with CD	Steroids + sulfasalazine	45,X/47, XY + 13 mosaicism
Knudtzon et al. (19), 1988	1 patient (diagnosis of CD at 21 years); fistulizing behavior	Prednisone, sulfasalazine, total proctocolectomy, and ileostomy	Mosaic pattern, 90% 46,X,i(Xq) and 10% 45,X
Manzione et al. (20), 1988	1 patient (20-year-old female) with UC	Hydrocortisone enemas and oral steroids during two exacerbations	46XdelX(p)
Lacaille et al. (21), 1995	1 patient (6-year-old female) with celiac disease, primary sclerosing cholangitis, and IBD	Sulfasalazine	45X0
Sokol et al. (24), 2002	1 patient (35-year-old female) with IBD and von Willerbrand disease, primary biliary cirrhosis	Olsalazine	45,X0
Durusu et al. (26), 2005	1 patient (29-year-old female) with celiac disease and CD	Prednisolone	45,X
Takaya et al. (27), 2006	1 patient (7-year-old female) with UC (from the rectum to the splenic flexure) and coarctation of the aorta	Mesalazine	45,X
Hyodo et al. (28), 2009	1 patient (13-year-old female) with UC	Mesalazine, dietary therapy, and steroids during the two recurrences	46,X,i(Xq)
Triantafillidis et al. (29), 2010	1 patient (24-year-old female) with autoimmune thyroiditis and CD (abscesses and fistula)	Infliximab (severe allergic reaction) and adalimumab	45X0
Keating et al. (33), 2020	1 patient (32-year-old female) with hypothyroidism, bicuspid aortic valve, CD	Budesonide, adalimumab (drug-induced cardiomyopathy), azathioprine, and ustekinumab	
Present case	1 patient (3-year-old female) with UC and atrial defect	Mesalazine, steroids, and azathioprine	45,X/46,XX

Nine cases (34%) were associated to other comorbidities such as autoimmune diseases (Hashimoto thyroiditis, primary biliary cirrhosis, acquired von Willebrand disease, celiac disease, and primary sclerosing cholangitis) and cardiac defects [bicuspid aortic valve, coarctation of the aorta, and atrial septal defect) (13, 17, 21, 24, 26, 27, 29, 33). Treatment with biologic drugs (infliximab, adalimumab, and ustekinumab) was reported in two cases, and in one case, a drug-induced cardiomyopathy during treatment with adalimumab was described (29, 33). Eight cases (31%) needed surgical treatments (four CD and four UC), and four of them died after surgery because of postoperative complications (cardiorespiratory arrest, hearth attack, and tetanus) (10–12, 14–16, 19).

The karyotype of the included cases was characterized by the absence of an X chromosome (monosomy or mosaic monosomy) in 12 cases (12, 14–16, 18, 21, 24, 26, 27, 29) or by the presence of a structurally abnormal X chromosome (or mosaic pattern with structurally abnormal X chromosome) in the remaining 13 cases (10, 11, 13–17, 19, 20, 28). In one case, the karyotype was not specified (33).

Three articles (a review and two observational studies) reported the prevalence rate of IBD in cohorts of TS without a clinical description of the cases (25, 30, 31). Overall, the prevalence of IBD in TS ranged from 0.67% to 4% (23, 25, 31, 32). One study, performed in Japan, described a 13-fold increase of the prevalence of IBD in women with TS compared to the general population (1.8 vs. 0.13%) with a difference more pronounced in CD (0.8 vs. 0.03%) compared to UC (1 vs. 0.1%) (32). The annual incidence of IBD in women with TS reported by one study was 270/100.000 (14). In another observational study, the standardized incidence ratio (SIR) of IBD in TS compared to the general population was 2.5 (95% CI 1.2–4.7) and 1.5 (95% CI 0.4–3.9) for UC and CD, respectively (30).

### Clinical Features and Prevalence of IBD in Down Syndrome

Ten articles reported cases of IBD in subjects with DS, adding up to a total of 18 cases, including our cases (12 with CD, 5 with UC, and 1 case reported as IBD) (34–43). Five (28%) were pediatric cases (36, 38, 39, 43). The three observational studies indicated a prevalence of IBD in cohorts of adults with DS ranging from 0.2 to 1.57% (35, 40, 41). Only one study compared data with a control population (subjects not affected by DS), describing adjusted rate ratios of 3.3 for UC (95%CI: 0.7–9.6) and 2.6 for CD (95%CI: 05–7.7) (40). Table 3 illustrates the characteristics of the published studies.

**Table 3:** Characteristics of published studies reporting cases of IBD in DS subjects.

**References**	**Publication year**	**Study design**	**Population (*n*)**	**IBD cases**	**Prevalence of IBD in DS**
Burgess et al. (34)	1971	Case report	1 subject	1 patient with CD	
Baccichetti et al. (35)	1990	Cross-sectional study from hospital registry	102 subjects with DS extracted from the registry of persons with disabilities of Belluno (Jan–Dec 1988)	1 patient with UC	Prevalence of IBD 0.9% (1/102)
Vajro et al. (36)	1998	Case report	1 subject	1 patient with CD	
Kaushik et al. (37)	2000	Case report	1 subject	1 patient with CD	
Persic et al. (38)	2001	Case report	1 subject	1 patient with CD	
Yamamoto et al. (39)	2002	Case report	1 subject	1 patient with CD	
Goldacre et al. (40)	2004	Cross-sectional case–control study from dataset of abstracts of hospital and death records	1,453 subjects with DS (mean age 13 years) and cohort of 460,000 people with other conditions for comparison (England 1963–1999)	3 patients with UC 3 patients with CD	Prevalence of UC and CD in the Down cohort: 0.2%. Adjusted rate ratios: 3.3 for UC (CI: 0.7–9.6) and 2.6 for CD (CI: 05–7.7)
Wallace et al. (41)	2007	Cross-sectional from hospital charts	57 subjects with DS (mean age: 37 years, DS: 13) (Brisbane, Australia Jan 2003–Mar 2005)	1 patient with IBD	Prevalence of IBD: 1.75% (1/57)
Souto-Rodríguez et al. (42)	2014	Case series	3 subjects	1 patient with UC 2 patients with CD	
Thaver et al. (43)	2016	Case report	1 subject	1 patient with CD	

Detailed clinical descriptions were available for nine cases only (36–39, 42, 43). Table 4 shows the clinical characteristics of the reported cases (including the case presented by our group). Two pediatric cases of CD were also affected by primary sclerosing cholangitis (PSC) (36, 37), one child had CD with pulmonary involvement (requiring infliximab and methotrexate) (43) and one adult woman with UC required colectomy at 34 years of age (42).

**Table 4:** Clinical characteristics of the cases of IBD and DS detailed in literature.

**Reference, year**	**Clinical characteristics**	**Treatment/ outcome**
Vajro et al. (36), 1998	1 male with PSC (diagnosis at 6 years) and CD (TI and right colon, diagnosis at 16 years)	Mesalazine and ursodeoxycholic acid
Kaushik et al. (37), 2000	1 patient with CD and PSC	Sulfasalazine and ursodeoxycholic acid
Persic et al. (38), 2001	1 female with CD, onset at 8 years (colonic CD), fistulizing behavior	Mesalazine, long-term steroids, and azathioprine
Yamamoto et al. (39), 2002	1 female with CD, diagnosis at 6 years and 5/12 (terminal ileum cecum and transverse colon)	Steroids + enteral and parenteral nutrition
Souto-Rodríguez et al. (42), 2014	One 34-year-old female with UC (diagnosed at 24 years) One 27-year-old male with CD (colon) One 35-year-old male with CD (right colon and ileocecal valve)	Mesalazine, steroids, and colectomy at 38 yearsMesalazineMesalazine and budesonide
Thaver et al. (43), 2016	1 female with CD, onset at 5 years (stomach, duodenum, and colon) with pulmonary involvement	Mesalazine, infliximab, and methotrexate (residual lung nodules)
Present case	One 10-year-old male with CD (colon, stomach, and esophageal stricture)	Enteral nutrition; azathioprine; several course of oral steroids, infliximab, and adalimumab; and endoscopic dilation

### Clinical Features and Prevalence of IBD in Other Genetic Syndromes

Twelve articles reported cases of IBD in subjects with other genetic syndromes (44–55). Seven papers reported eight cases of IBD (three CD and five UC) in patients with NF1 (44–50), with two pediatric UC cases (48, 49). Table 5 illustrates the characteristics and results of these studies. Cases of IBD in NF1 were not described as particularly severe, and no lethal cases were reported. Two articles described two cases of IBD in subjects with DGS (an 8-year-old boy and a 17-year-old boy), treated with biological drugs (vedolizumab and infliximab) (54, 55). In literature, two cases with KS and IBD were reported (one child with CD and the other case was not detailed) (52, 53) and one case (a 20-year-old female) with WS and CD, subjected to medical treatment, was also described (51).

**Table 5:** Characteristics and main results of studies on NF1 and IBD.

**References**	**Publication year**	**Study design**	**Case report**	**Treatment**
Henderson et al. (44)	1985	Case report	1 patient (30-year-old female) with NF1, CD, osteopetrosis, and Paget's disease	Azathioprine and prednisolone enemas
Cameron et al. (45)	1989	Case reports	1 patient (57-year-old female) with NF1 and UC 1 patient (29-year-old female) with NF1 and CD	Sulfasalazine and rectal corticosteroid therapyCorticosteroid therapy and sulfasalazine
Tavakkoli et al. (46)	2009	Case report	1 patient (39-year-old female) with NF1 and UC	Corticosteroid therapy and sulfasalazine
Triantafillidis et al. (47)	2009	Case report	1 patient (56-year-old male) with NF1 and CD Colectomy complicated by thrombosis of the right subclavicular vein	Steroids, mesalazine, and right hemicolectomy
Baratelli et al. (48)	2014	Case report	1 patient (14-year-old male) with NF1 and UC	Mesalazine
Adams et al. (49)	2016	Case report	1 patient (15-year-old male) with NF1 and UC	Oral prednisone, mesalazine, and omeprazole
Fukunaga et al. (50)	2017	Case report	1 patient (34-year-old female) with NF1 and UC	Mesalazine and prednisolone

## Discussion

With this systematic review, we aimed to characterize the clinical features and epidemiological aspects of IBD in specific genetic syndromes. Following our inclusion criteria, we identified a total of 69 cases of IBD (considering our two unpublished cases) described in six different genetic syndromes, including DS, TS, NF1, DGS, WS, and KS. DS and TS are conditions related to chromosomal abnormalities (21 and X chromosome, respectively), DGS and WS are chromosomal deletion syndromes (22q and 7q11.23, respectively), while NF1 and KS are related to single-gene mutations (NF1 and MLL2 or KDM6A for NF1 and KS, respectively). All of these are genetic diseases associated with a known risk of autoimmune comorbidities, with the only exception being represented by the NF1, where this association has been rarely described (56–59). The presence of an immunological dysfunction has been widely documented in DS, TS, KS, WS, and DGS. Although the association between these genetic conditions and IBD is known, many confounding factors can delay this diagnosis. In fact, all these syndromes share other relevant features, such as the presence of poor growth or short stature (on a genetic basis) and gastrointestinal symptoms (due to gastrointestinal malformations or comorbidities) that overlap with the main symptoms of IBD. Additionally, a variable degree of intellectual disability is reported in all these syndromes, complicating the symptom reporting and their correct assessment. Therefore, it can be difficult to maintain a high index of suspicion of IBD in this particular scenario, with a possible misdiagnosis or a diagnostic delay. Furthermore, the presence of other comorbidities in all these genetic conditions (e.g., cardiac, renal, hematological, and immunological) can complicate the course of IBD, making the patient more vulnerable to developing drug toxicities, infections, and surgical complications and higher risk of mortality (Figure 1).

The results of our systematic search show that the majority of cases of IBD were described in subjects with TS (38 cases including our novel case). Ten cases were particularly severe, with eight requiring surgery and two treated with biologics. It should be noted that all cases with surgical complications or with a lethal outcome and 50% of cases of TS and IBD were published before the year 2000, when biological treatment was rarely used in IBD. In the biologic era, no mortality for IBD has been recorded in females with TS. No early-onset IBD (before 5 years of age) was reported, apart from our case. We could then speculate that TS is not associated to a particularly severe phenotype of IBD; however, no comparison data with the general IBD population (not affected by TS) is available so far. Interestingly, a case of cardiomyopathy induced by adalimumab in a TS patient with bicuspid aortic valve has been recently presented, suggesting caution when using anti-TNF alpha in this fragile category of patients (33).

Epidemiological data for TS were extracted from six studies. Both prevalence (according to five studies) and incidence (based on two reports) indicated a higher risk of IBD in females with TS compared to the general population. These data undoubtedly indicate the need for a gastroenterological follow-up in subjects with TS.

The link between TS and autoimmune or inflammatory disorders could be mediated by genes located on the X-chromosome (e.g., the MHC-paralogs and the Toll-like receptor 8 signaling) and hormonal imbalance (characterized by elevated FSH and LH) that can activate immune pathogenic mechanisms. Based on a previous review of literature including a total of 15 cases of IBD in TS, it has been suggested that females with the isochromosome Xq (found in 60% of the women with IBD and TS vs. 17% of TS women without IBD) are more susceptible to IBD compared to those with the monosomy X (22). Our review, based on 25 genetically confirmed cases, does not reiterate this data, indicating an equal distribution of monosomy and structurally abnormal X chromosome.

Even if cases of IBD and DS were less frequently reported in literature, with a total of 13 adults and 5 children described (including our case), prevalence data (retrospectively collected) from two cross-sectional studies and from one cross-sectional controlled study show a two- to three-fold increase in the prevalence of IBD in DS compared to the general population (35, 40, 41). No longitudinal study was available, with a lack of data on the incidence of IBD in cohorts of subjects with DS. Plausible reasons for an increased risk of autoimmune disorders in DS include the following: involvement of genes on chromosome 21 implicated in the immune response (such as interferon receptor, CD 18, or the gene AIRE), defective T-cell control mechanisms and a reduced expansion of T-cell precursors in the thymus, a higher representation of immature NK cells with lower intrinsic activity, reduced IL-2 expression, defective phagocyte chemotaxis, and oxygen radical production (55).

Besides our pediatric case, one other case described in literature was particularly complicated due to the pulmonary involvement, which is a very rare complication of CD (43). Our case was challenging for several reasons, specifically, 1. the presence of a previously unrecognized esophageal stricture and a long-standing history of dysphagia with solid food refusal and 2. the poor response to standard treatments. Esophageal involvement in CD is uncommon, and its presentation with an esophageal stricture is even less common. The most frequent symptoms are dysphagia and odynophagia, and a large number of patients (up to 1/3) also have oral lesions. Esophageal CD can present as an erosive–ulcerative esophagitis or with an esophageal stricture (generally in the medium or distal esophagus) or fistula. Histology can be unspecific, adding on diagnostic difficulties (60, 61). Differential diagnoses include congenital stenosis or post esophageal atresia repair, gastroesophageal reflux disease, eosinophilic esophagitis, unknown caustic ingestion, and few other rarer causes. All of these conditions should be investigated in patients with DS and esophageal stricture, considering the high incidence of congenital gastrointestinal malformations, gastrointestinal reflux, and intellectual disability (with possible unrecorded foreign bodies or caustic ingestion) (62). In our case, the proximal esophageal stricture was considered compatible with CD, although histology was not specific and the response to anti-inflammatory treatments was poor, requiring endoscopic dilation. Even if there was no history, an origin related to a previous ingestion of caustics of foreign body cannot completely be excluded in our case, considering the severe intellectual disability, equally a congenital origin.

According to the current CD guidelines (63), our patient was considered at high risk at diagnosis; therefore, upfront infliximab was chosen as induction treatment along with EEN. An immunomodulator (azathioprine) was introduced at the first relapse (3 months after diagnosis) and, despite the optimization of infliximab schedule (10 mg/kg every 4 weeks) and the combination with azathioprine, the response was unsatisfactory with several course of steroids required during the follow-up and the necessity to switch to adalimumab. In other cases of IBD in DS reported in literature, response to standard treatments is described as good, except for one case of UC where colectomy was required and the CD case with pulmonary involvement that was treated with infliximab and methotrexate. Patients with DS are generally prone to drug side effects; this is frequently reported with some antiblastic agents, and toxicities (including cardiotoxicity, myelosuppression, infections, and mucositis) have a multifactorial origin (64–67). The greater risk of side effects with high doses of methotrexate in acute leukemia in patients with DS is a typical example; therefore, specific protocols with dose reduction for subjects with DS are currently used (68–71). In the same subjects, a higher accumulation of thioguanine inside erythrocytes in comparison to non-DS children has also been described (72). No specific reports of toxicities related to IBD medications (including thiopurines, methotrexate, and anti-TNF-α) in subjects with DS have been published so far. The risk of malignancy is another concern dealing with DS. Trisomy 21 is associated with a 10- to 20-fold risk for developing acute lymphoblastic leukemia (ALL) and acute myeloid leukemia (AML), as compared to non-DS children, while the risk of developing solid tumors is not increased (73, 74). Long-term use of some IBD medications (mainly thiopurines with or without anti TNF-α) has been associated to an increased risk of malignancy, particularly a rare form of lymphoma (hepatosplenic T-cell lymphoma) (75–79). In the absence of specific data on toxicity and carcinogenesis of IBD medications in subjects with DS, caution and close clinical follow-up is recommended, particularly with drugs such as azathioprine, methotrexate, and anti-TNF-α.

Lastly, the systematic research identified sporadic cases of IBD in other genetic syndromes. Interestingly, eight cases (six adults and two children), five with UC, have been described in NF1 with five cases reported since 2009. Unfortunately, there are no longitudinal or controlled studies exploring the link between NF1 and IBD, but as more cases accumulate, a correlation beyond coincidence becomes likely. NF1 is one of the RASopathies, a group of genetic syndromes due to germline mutations in genes that encode protein components of the RAS–mitogen-activated protein kinase (MAPK) pathway. RAS has an essential role in adaptive immunity and normal immune cell function, and the MAPK is crucial in the process of inflammation and apoptosis (80). Activated MAPK pathway has been reported previously in acetic acid-induced colitis (81, 82), and MAPK inhibitors were found to reduce inflammation (83) and specifically to ameliorate colitis in animal models of IBD (84, 85). These evidences can explain the basis for a susceptibility to IBD in patients with NF1. Furthermore, an altered mast cell function has been advocated as a possible common mechanism in the pathogenesis of both NF1 and IBD (particularly UC) and could be a link between the two conditions (86–90). From the systematic review, there is no evidence of an enhanced severity of these cases; however, NF1 is another condition associated to the risk of malignancy; therefore, a particular attention to the treatment choice should be reserved to these patients.

Despite the rigorous methodology followed and the extensive review (including several genetic syndromes), this systematic review has some limitations. The major limitations are related to the quality of the studies under review and the small number of available studies. Our results were necessarily based on case reports and series and few cohort studies; as a result, it was not possible to perform a meta-analysis. These limitations further highlight the need for the following: 1. population-based epidemiological studies for specific syndromes focused on the development of IBD and 2. specific studies or registry data sub-analysis investigating clinical characteristics, outcomes, and drug toxicities in subjects with IBD and a genetic syndrome compared to subjects with IBD without an underlying genetic condition.

In conclusion, we found evidence of IBD in several genetic syndromes, with most of the reported cases in TS and DS. Literature on the coexistence of NF1 and IBD has been accumulating in the last years, suggesting a possible link between these two conditions. Both the prevalence and incidence of IBD seem to be increased in subjects with TS and DS compared to the general population, although there is a paucity of high-quality and longitudinal epidemiological data. Patients with IBD and a genetic syndrome require a particular attention during the follow-up, considering the possible association with gastrointestinal and extra-intestinal comorbidities. A careful choice of the immunosuppressive regimens is necessary, particularly in DS, relating to the high risk of malignancy and the enhanced susceptibility to drug side effects, and the development of targeted protocols is required.

## Data Availability Statement

The original contributions presented in the study are included in the article/supplementary material, further inquiries can be directed to the corresponding author/s.

## Author Contributions

SG and CC designed the study. SG and GG performed literature search and review, wrote the manuscript, and critically revised it. SG, GG, GC, and CC critically reviewed the manuscript. All authors contributed to the article and approved the submitted version.

## Conflict of Interest

The authors declare that the research was conducted in the absence of any commercial or financial relationships that could be construed as a potential conflict of interest.

## Publisher's Note

All claims expressed in this article are solely those of the authors and do not necessarily represent those of their affiliated organizations, or those of the publisher, the editors and the reviewers. Any product that may be evaluated in this article, or claim that may be made by its manufacturer, is not guaranteed or endorsed by the publisher.
